# Rare subcommunity maintains the stability of ecosystem multifunctionality by deterministic assembly processes in subtropical estuaries

**DOI:** 10.3389/fmicb.2024.1365546

**Published:** 2024-04-19

**Authors:** Shu Yang, Qinghua Hou, Nan Li, Pengbin Wang, Huaxian Zhao, Qingxiang Chen, Xinyi Qin, Jiongqing Huang, Xiaoli Li, Nengjian Liao, Gonglingxia Jiang, Ke Dong, Tianyu Zhang

**Affiliations:** ^1^Key Laboratory of Climate, Resources and Environment in Continental Shelf Sea and Deep Sea of Department of Education of Guangdong Province, Department of Oceanography, Key Laboratory for Coastal Ocean Variation and Disaster Prediction, College of Ocean and Meteorology, Guangdong Ocean University, Zhanjiang, China; ^2^Key Laboratory of Environment Change and Resources Use in Beibu Gulf, Ministry of Education (Nanning Normal University), Nanning, China; ^3^Key Laboratory of Marine Ecosystem Dynamics, Second Institute of Oceanography, Ministry of Natural Re-sources, Hangzhou, China; ^4^School of Agriculture, Ludong University, Yantai, China; ^5^College of Environmental Science and Engineering, Guilin University of Technology, Guilin, China; ^6^Department of Biological Sciences, Kyonggi University, Gwanggyosan-ro, Yeongtong-gu, Suwon-si, Republic of Korea

**Keywords:** 16S rRNA, biodiversity, community assembly, ecosystem multifunctionality, estuary ecosystem, rare tax

## Abstract

Microorganisms, especially rare microbial species, are crucial in estuarine ecosystems for driving biogeochemical processes and preserving biodiversity. However, the understanding of the links between ecosystem multifunctionality (EMF) and the diversity of rare bacterial taxa in estuary ecosystems remains limited. Employing high-throughput sequencing and a variety of statistical methods, we assessed the diversities and assembly process of abundant and rare bacterioplankton and their contributions to EMF in a subtropical estuary. Taxonomic analysis revealed Proteobacteria as the predominant phylum among both abundant and rare bacterial taxa. Notably, rare taxa demonstrated significantly higher taxonomic diversity and a larger species pool than abundant taxa. Additionally, our findings highlighted that deterministic assembly processes predominantly shape microbial communities, with heterogeneous selection exerting a stronger influence on rare taxa. Further analysis reveals that rare bacterial beta-diversity significantly impacts to EMF, whereas alpha diversity did not. The partial least squares path modeling (PLS-PM) analysis demonstrated that the beta diversity of abundant and rare taxa, as the main biotic factor, directly affected EMF, while temperature and total organic carbon (TOC) were additional key factors to determine the relationship between beta diversity and EMF. These findings advance our understanding of the distribution features and ecological knowledge of the abundant and rare taxa in EMF in subtropical estuaries, and provide a reference for exploring the multifunctionality of different biospheres in aquatic environments.

## 1 Introduction

Microorganisms are the important drivers of biogeochemical cycles in the ocean environment and have vital roles in affecting global nutrient cycles and maintaining ecosystem multifunctionality (EMF) (Falkowski et al., 2008; Strom, 2008; Madsen, 2011; Alsterberg et al., 2017; Villnäs et al., 2018). Over decades, a growing number of studies have shown that the diversity of individual groups and EMF are closely related (Duffy, 2009; Snelgrove et al., 2014; Delgado-Baquerizo et al., 2016; Luo et al., 2018; Hu et al., 2021). For instance, Delgado-Baquerizo et al. (2016) found that EMF increased with soil bacterial alpha diversity in global drylands. Luo et al. (2018) investigated the link between microbial diversity and EMF in long-term fertilized agricultural soil and the results reveal a positive correlation between soil microbial diversity and EMF. However, the associations among species diversity, phylogenetic diversity and EMF in the natural environment remain unclear.

Highly abundant species can inhabit distinct niches, competitively exploit a range of various resources, and well adapt to environmental changes (Zhang et al., 2022), and their importance in driving ecosystem functioning has been recognized. Accumulating evidence has demonstrated that rare taxa have a disproportionate impact on EMF that in disproportionate to its abundance (Winfree et al., 2015; Banerjee et al., 2018; Wan et al., 2021; Zhang et al., 2022). For example, Zhang et al. (2022) found that, in comparison to abundant species, the diversity of rare species showed a greater positive relationship with EMF. These studies indicate that the rare taxa diversity appears to be a promising indicator for assessing EMF.

The assembly processes of microbial communities directly influence microbial diversity, include species abundance and a multitude of functions. Accumulating evidence demonstrates that the assembly process can be understood through both stochastic and deterministic processes. Given the distinct environmental gradients in estuarine ecosystems, microbial communities encounter varying degrees of environmental stress, which results in distinct assembly patterns of planktonic bacterial communities (McLusky and Elliott, 2007). Yao et al. have clarified these dynamics, revealing that homogeneous selection (deterministic process) significantly shapes bacterial community assembly across the estuarine marsh ecosystem (Yao et al., 2019). Additionally, Isabwe et al. found longitudinal distribution patterns of bacterioplankton in riverine ecosystems, suggesting that distance-decay relationships may be attributed to ecological drift (stochastic process) (Isabwe et al., 2022). Nonetheless, the assembly processes of both abundant and rare biospheres in influencing arestill not well comprehended in estuarine ecosystems.

Estuary ecosystems are the most ecosystem functionality systems on earth and have an essential role in biogeochemical cycles (Guo et al., 2015; Wang and Zhang, 2020). Sanniang Bay is located in the Beibu Gulf. Therefore, to explore the significance of abundant and rare taxa on EMF under environmental disturbance, we analyzed two years of seasonal water samples in a subtropical estuary to (1) identify the abundant and rare taxa across environmental disturbance of the subtropical estuary; (2) reveal the assembly process of the abundant and rare subcommunities; (3) investigate the contribution of abundant/rare taxa to biodiversity and EMF; and (4) identify the key drivers affecting EMF via abundant/rare subcommunity.

## 2 Materials and methods

### 2.1 Sampling stations and environmental parameters

The sites were located in the estuary of Dafeng River and Sanniang Bay, Guangxi Zhuang Autonomous Region, China (Supplementary Figure S1). In 2018, surface water samples (0.5 meters) were collected quintuplicate samples from four seasons at 15 sites (Supplementary Figure S1). In 2020, samples were collected from 16 sites (Supplementary Figure S1). We employed five-point sampling method to collected surface water samples using a rosette of Niskin bottles at each designated site. In 2018, we were unable to obtain the S8 samples, resulting in the collection of a total of 620 samples. We roughly divide the sampling location into three sub-areas: a low salinity (LS) group, a medium salinity (MS) group and a high salinity (HS) group. To analyze nutrients and chlorophyll a, for each sample, 500 mL seawater were filtered through a 0.45 μm filter (Millipore Corporation, Billerica, MA). Until further analysis, the filters were kept in storage at −80°C. The environmental factors were measured by our previous methods (Zhao et al., 2023) and all of the environmental factors are listed in Supplementary Table S1.

### 2.2 DNA extraction and PCR amplification

Two liters of each surface water sample for microbial diversity were filtered through 0.22 μm membranes according to a previous study (Teeling et al., 2012), DNA quality was assessed using a spectrophotometer (Delaware, United States). DNA samples were stored at −80°C. According to a previous publication (Zhao et al., 2023), we used the universal primers 341F (CCTACGGGNGGCWGCAG) and 805R (GACTACHVGGGTATCTAATCC) for 16S rRNA amplification (Herlemann et al., 2011). The 50 μL PCR mixture containing 2 μL of template DNA, 25 μL of 2 × Taq PCR MasterMix (TianGen), 21 μL of PCR grade molecular water and 1 μL of each primer was aliquoted into PCR Beads. PCR (50 μL) was conducted on a Biorad thermocycler, and the conditions were as follows: 60 s initial denaturation at 95°C, 34 cycles of 30 s denaturation at 95°C, 30 s annealing at 54°C, 60 s elongations at 72°C. A TruSeq DNA kit (Illumina, United States) was used to processing PCR products for sequencing (Zhao et al., 2023).

### 2.3 High-throughput sequencing and taxonomic annotation

The libraries were prepared following the Illumina library preparation protocols. The library was delivered to Majorbio Co. Ltd. (Shanghai, China) for Illumina MiSeq platform (PE300, 2 × 300 bp) for sequencing. Employing the DADA2 denoising method (Caporaso et al., 2010; Callahan et al., 2017), reads with quality scores below 100, as well as chimeric sequences, barcode sequences, and primers, were systematically removed. The amplicon sequence variants (ASVs) obtained from the raw data were selected for further analysis (Callahan et al., 2017). The Ribosomal Database Project Classifier program was picked to perform taxonomic assignment at 80% confidence level (Maidak et al., 2000). The sequence data were deposited in GenBank at BioProject Accession: PRJNA971362.

### 2.4 Calculation of ecosystem multifunctionality

To obtain the multi-nutrient cycling index (MNI), we first normalized and standardized each of the relevant factors linked to the cycling of phosphorous (TP, DIP), nitrogen (NO_3_^−^-N, NO_2_^−^-N, NH_4_^+^-N, DIN, TN) and carbon elements (TOC) using the *Z*-score transformation method. These standardized ecosystem function values ranging from 0 to 1 were computed using the formula:

$$STD=\frac{\left(V-V_{\text{min}}\right)}{\left(V_{\text{max}}-V_{\text{min}}\right)}$$

*V* represents the value of chosen factors, where *V*_min_ is the minimum value and *V*_max_ is the maximum value of these selected factors. The mean value of these calculating results represents the MNI index (Delgado-Baquerizo et al., 2016; Zhang et al., 2021).

### 2.5 Statistical analysis

To reduce bias caused by differences in sequencing depths, the samples in this study were rarefied to the minimum sample depth required for further analysis (Feng et al., 2022). In this study, ASVs were classified into abundant (>0.1%), rare (<0.01%) and intermediate (0.01–0.1%) (Jiao and Lu, 2020; Zhao et al., 2023). We calculated the diversity indices (Kemp and Aller, 2004) and employed the Shannon index to represent alpha diversity and the richness index to reflect the species pool (Supplementary Table S2). Beta diversity was quantified by Bray–Curtis distance, and the comparison of groups was used permutational multivariate analysis of variance (PERMANOVA). Partial least-squares path modelling (PLS-PM) analysis was performed by ‘plspm’ R package. Following the methodology outlined by Stegen et al. (2013), a null model analysis was performed to classify community assembly processes. This analysis involved generating beta-diversity metrics, including the beta-nearest taxon index (βNTI) and Bray–Curtis based Raup-Crick (RC_bray_), to evaluate community assembly from both phylogenetic and taxonomic perspectives. Results indicating |βNTI| >2 were interpreted as predominance of deterministic process, while |βNTI| <2 suggested a stochastic process dominance. Further analysis on pairwise comparisons with |βNTI| <2 using RC_bray_ values revealed the roles of dispersal limitation with drift, and homogenizing dispersal with drift, as indicated by RC_bray_ values >+0.95, <−0.95, and <+0.95, respectively. The Spearman’s rank method was used to calculate correlations for the partial Mantel and Mantel test. The R package ‘ggplot2’ was used to conduct a linear regression plot analysis.

## 3 Results

### 3.1 Composition and diversity of the abundant and rare taxa

**Figure 1:**
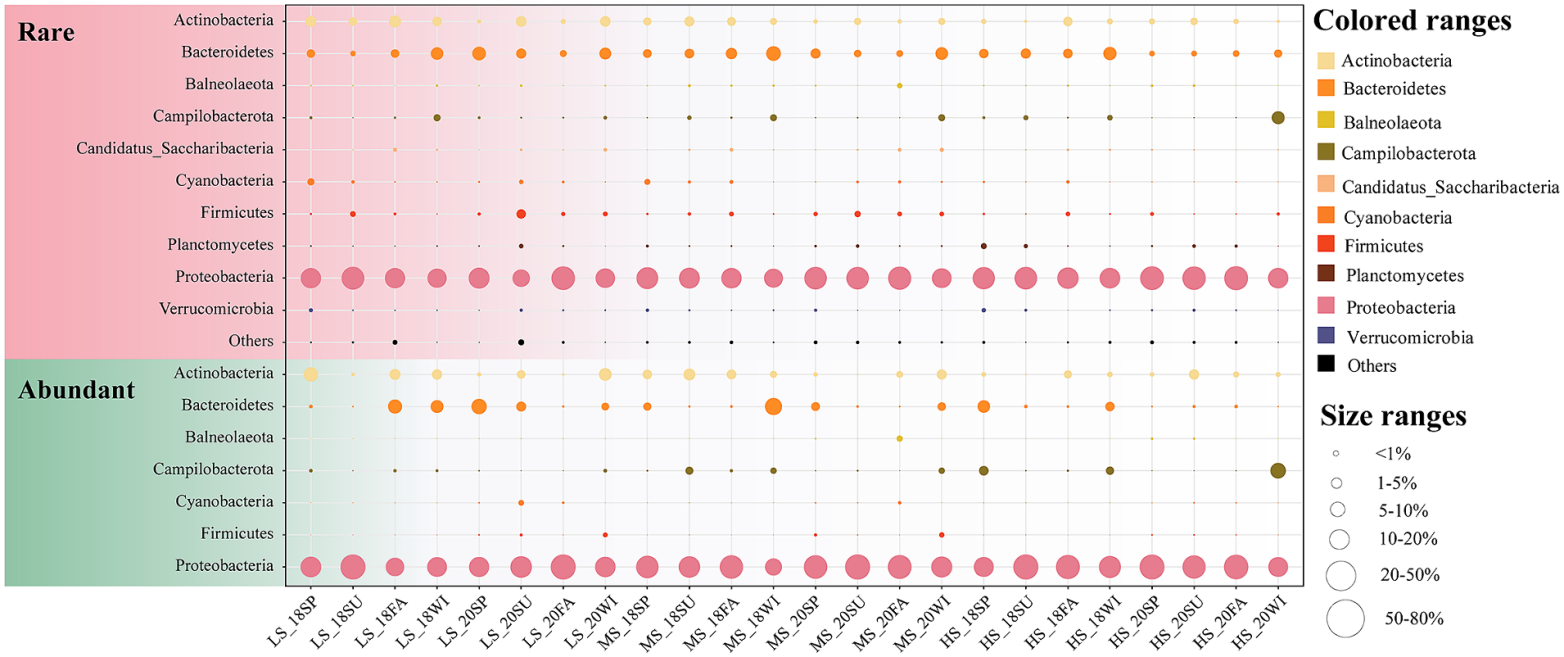
Marine bacterioplankton distribution and abundance of total, rare and abundant subcommunities from different seasons and salinity gradients. Circle size indicates species abundance. LS, low salinity; MS, medium salinity; HS, high salinity: SP, Spring; SU, Summer; FA, Fall; WI, Winter.

In this study, 620 surface water samples across different seasons were collected from Sanniang Bay (Supplementary Figure S1). Ultimately, a total of 39,969,028 high quality sequences were obtained, which belonged to 2,454 ASVs. A total of 120 ASVs (4.89%) with 31,318,993 (78.36%) sequences were classified as abundant ASVs, while 1774 ASVs (72.29%) with 2,731,363 (6.83%) sequences were classified as rare ASVs. Proteobacteria was the dominant phylum in the abundant (84.99%) subcommunity, followed by Actinobacteria (5.59%) and Bacteroidetes (5.28%), with the most prevalent classes in the abundant subcommunity being Alphaproteobacteria (50.02%) and Gammaproteobacteria (32.81%). The rare subcommunity was mainly composed of Proteobacteria (70.92%), followed by Bacteroidetes (11.24%) and Actinobacteria (4.42%), with Alphaproteobacteria (30.73%) and Gammaproteobacteria (30.33%) being the most dominant classes (Figure 1). Diversity analysis indicated that rare taxa exhibited significantly greater alpha diversity than abundant taxa. Within the abundant subcommunity, bacterioplankton communities in the LS group exhibited significant temporal variation (*p* < 0.05). For example, the alpha diversity of the LS group showed an upward trend from spring to winter and peaked in winter 2020 (Supplementary Figure S2). In contrast, the alpha diversity of rare taxa exhibited more dramatic interannual variation in the HS samples than in the LS and MS samples (Supplementary Figure S2). In the rare subcommunity of HS samples, the lowest alpha diversity was observed in summer 2018, and the highest was observed in fall 2020 (Supplementary Figure S2). Species pool analysis revealed that the species pool was smallest in summer 2018 and largest in fall 2020 in the rare taxa. In the abundant subcommunity, the smallest species pool was observed in the spring HS samples of 2018, and the largest was observed in the fall LS samples of 2020 (Supplementary Figure S2). The results of the PERMANOVA revealed a more significant difference in beta diversity within sites for communities composed of abundant subcommunities (*r*^2^ = 0.030, *p* = 0.001) compared to rare subcommunities (*r*^2^ = 0.027, *p* = 0.001) (Supplementary Table S3).

### 3.2 Assembly process of abundant and rare taxa

**Figure 2:**
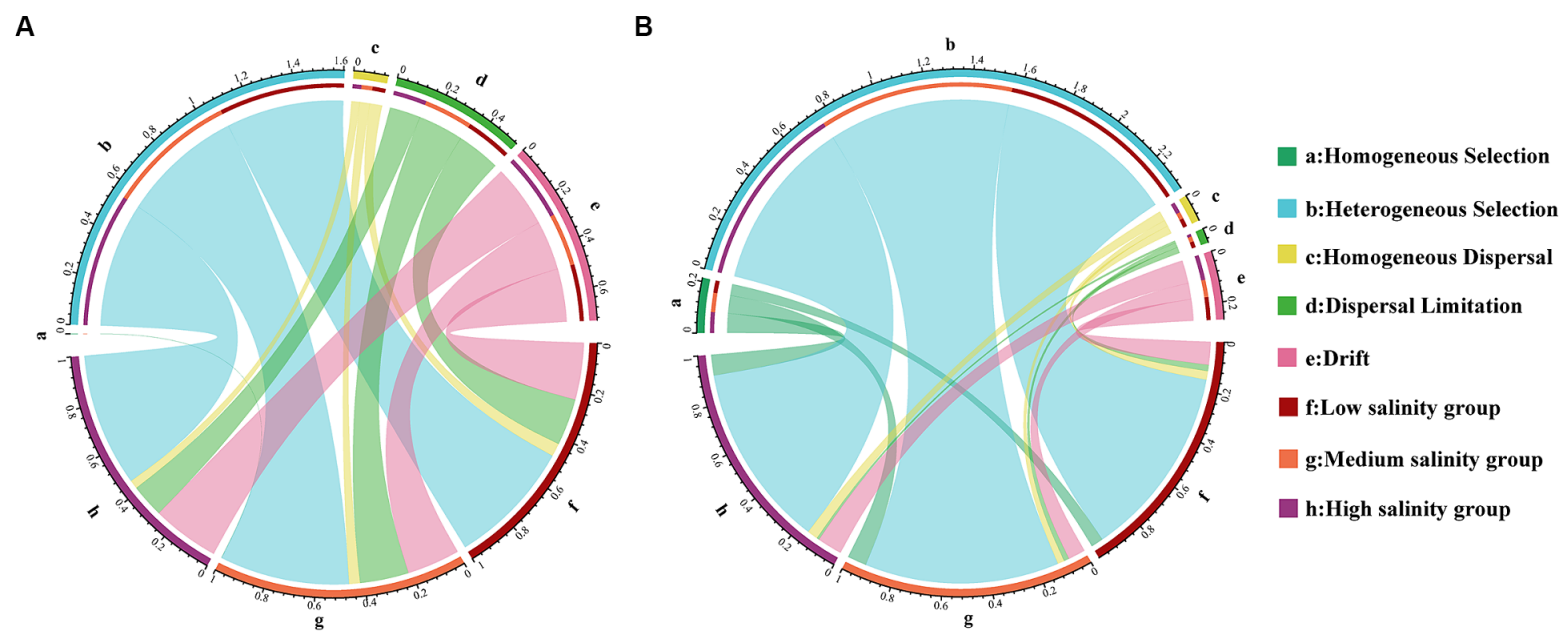
Analysis of the community assembly of marine bacterioplankton with abundant **(A)** and rare **(B)** marine bacterioplankton communities displayed by Circos analysis.

A statistical technique using βNTI was used in this study to decipher the community assembly processes. The βNTI values between >2 or <−2 were 53.90 and 86.27% for the bacterial community in abundant and rare taxa, respectively. The results show that deterministic processes had a crucial role in the assembly of different biospheres than stochastic processes. We observed greater heterogeneous selection in the rare taxa. Within the community of abundant taxa, heterogeneous selection was most pronounced in the MS group (29.7%) and least evident in the HS group (13.5%). For rare taxa, heterogeneous selection was most significant in the HS group, reaching 55.4%, and least pronounced in the LS group, at 48.9% (Figure 2). Interestingly, the heterogeneous selection of abundant taxa increases with higher salinity concentrations, while rare taxa do not (Figure 2). Within the stochastic process, it was drift that predominantly influenced the assembly of abundant and rare taxa (Figure 2).

### 3.3 Correlation between different taxa diversity and EMF

**Figure 3:**
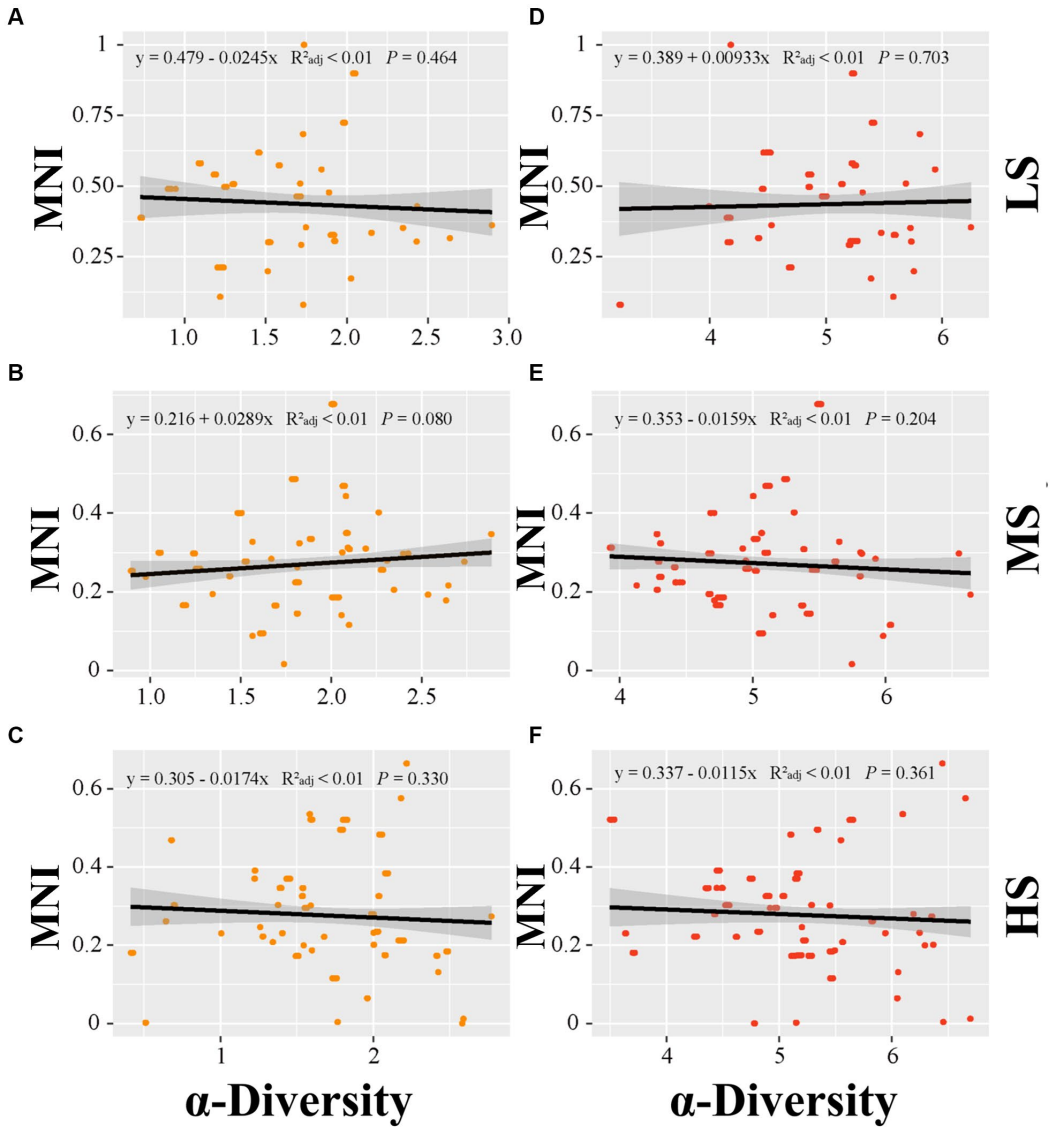
Linear regressions for α-diversity of abundant **(A–C)** and α-diversity of rare marine bacterioplankton communities **(D–F)** with EMF in different salinity gradients. LS, low salinity; MS, medium salinity; HS, high salinity; MNI, multi-nutrients cycling index.

**Figure 4:**
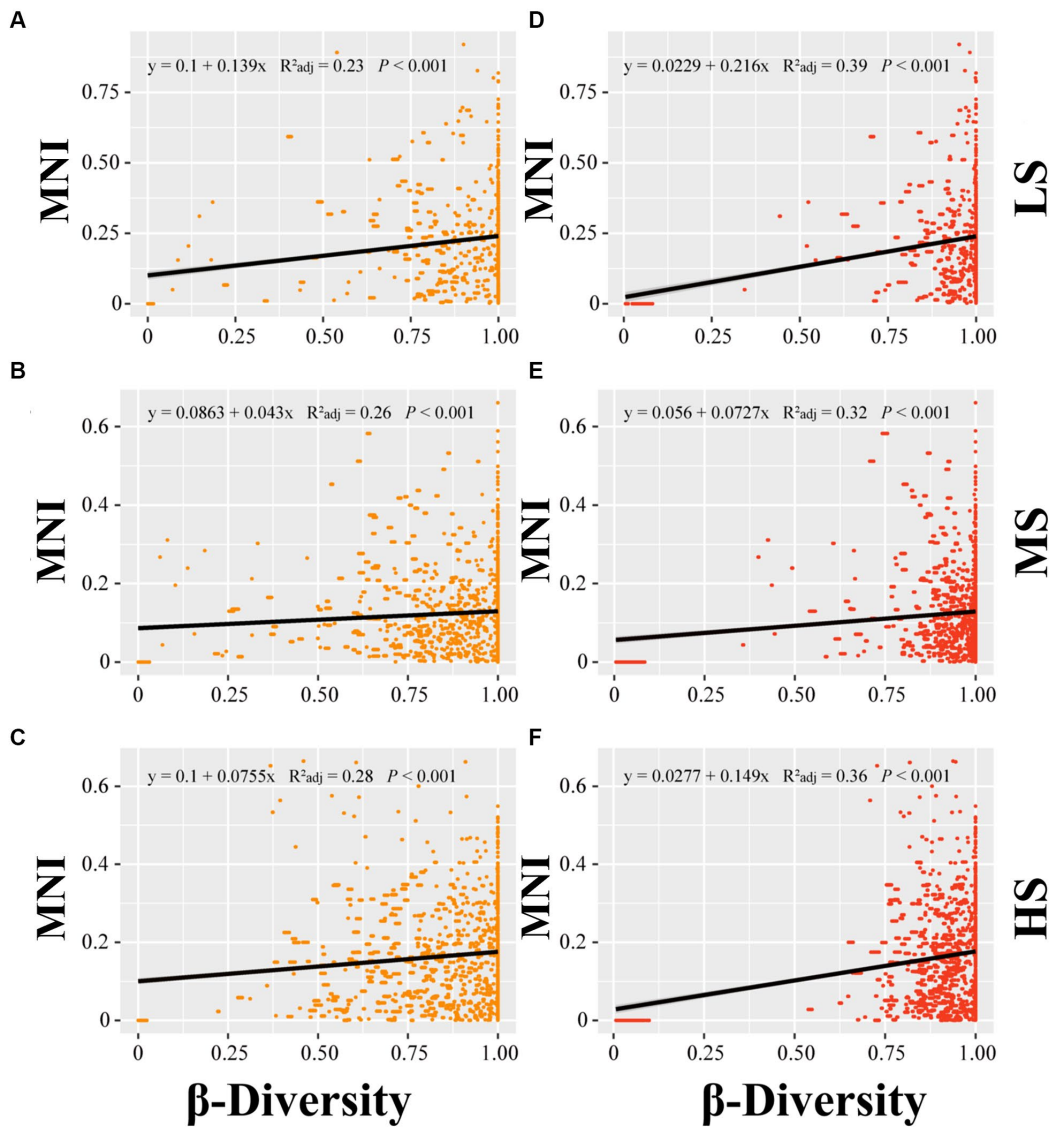
Linear regressions for β-diversity of abundant **(A–C)** and β-diversity of rare **(D–F)** marine bacterioplankton communities with EMF in different salinity gradients. LS, low salinity; MS, medium salinity; HS, high salinity; MNI, multi-nutrients cycling index.

**Figure 5:**
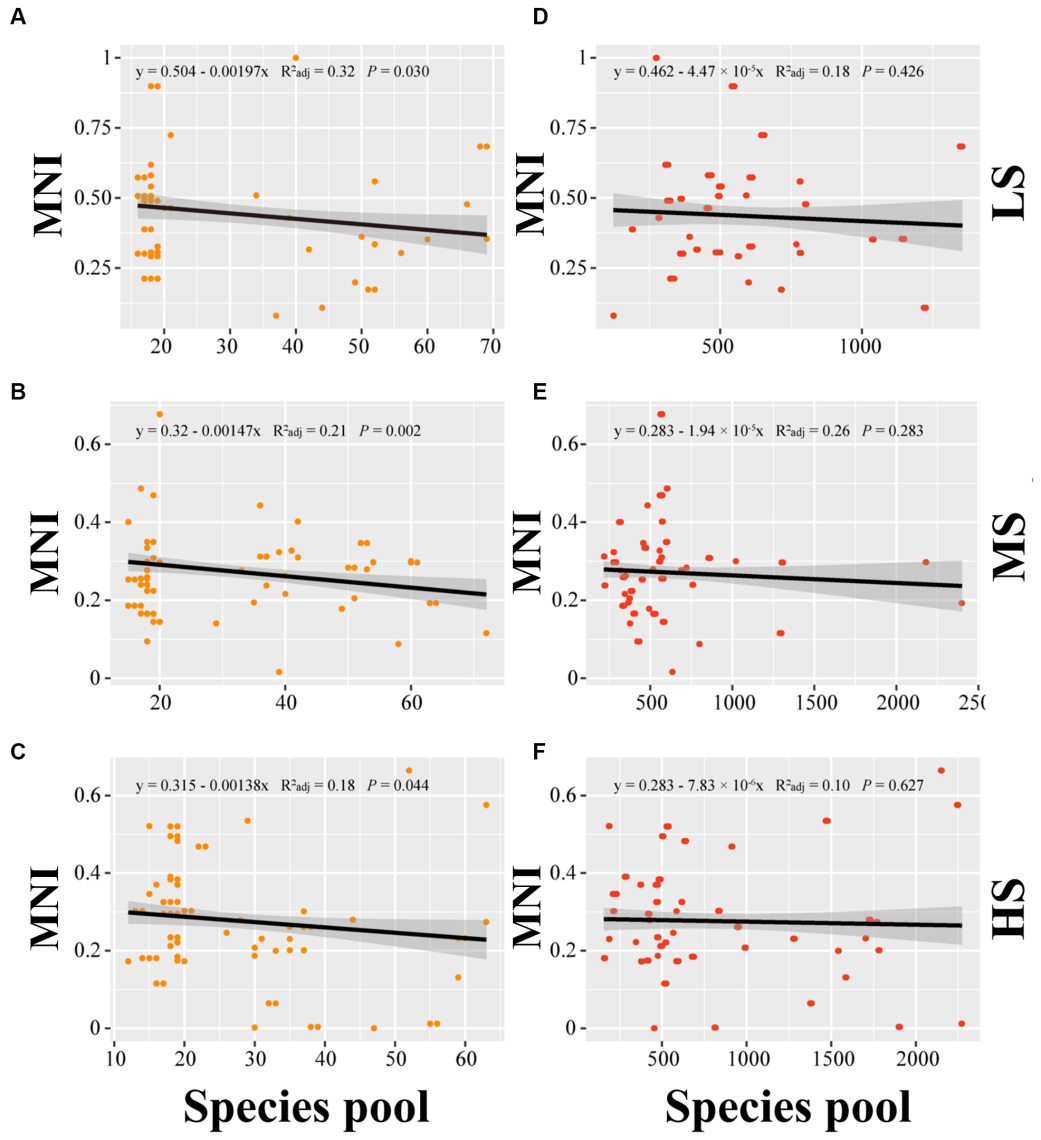
Linear regressions for the species pools of abundant **(A–C)** and species pools of rare **(D–F)** marine bacterioplankton communities with EMF in different salinity gradients. LS, low salinity; MS, medium salinity; HS, high salinity; MNI, multi-nutrients cycling index.

Linear regression analysis was employed to establish the relationship of MNI to alpha diversity, beta diversity and species pools in abundant and rare areas. In terms of alpha diversity, the linear regression analysis indicated no significant correlation between alpha diversity and MNI in abundant and rare taxa (*p* > 0.05) (Figure 3). For beta diversity, the linear regression analysis demonstrated that MNI showed a significantly positive correlation with beta diversity of abundant and rare taxa in LS, MS, and HS samples (*p* < 0.001). Compared to abundant taxa, the beta diversity of rare taxa showed a stronger influence on the EMF (Figure 4). Moreover, compared with MS and HS samples, the beta diversity of rare taxa in the LS group had a stronger linear correlation with MNI (Figure 4). The results in Figure 5 show that MNI was significantly negatively correlated with the species pool in the LS, MS, and HS samples in the abundant subcommunity (*p* < 0.05). However, there was no significant relationship between the species pool and MNI in the LS, MS, and HS samples of rare taxa (*p* > 0.05) (Figure 5).

### 3.4 Environment factor effects on microbial biosphere diversity and EMF in subtropical estuary ecosystems

In this study, we utilized Spearman analysis to evaluate the impact of individual environmental factors on alpha diversity and the species pool of abundant and rare taxa. The association between alpha diversity and environmental factors varied across different groups (Supplementary Table S4). In the abundant subcommunity, a significant positive link was found between TOC and pH with alpha diversity. TOC exerted the most significant positive influence in the LS group, while NH_4_^+^-N and DIP were found to have significant negative corrections with alpha diversity in the MS and HS samples, respectively (Supplementary Table S4). In a rare subcommunity, the two most significant factors affecting alpha diversity and species pool in the LS and HS groups were found to be DO (positive) and DIP (negative). Additionally, TP was identified as another important but negatively influencing factor for the HS group. Among the numerous influencing factors in the MS group, temperature was the predominant positive factor shaping alpha diversity and species pool, while Chl-*a* had a negative influence on alpha diversity and species pool (Supplementary Table S4). The Mantel test results were used to further analyse the effect of distinct environmental factors on beta diversity and community assembly mechanism (βNTI) of different microbial biosphere species. In the abundant subcommunity, beta diversity was influenced by different environmental factors along salinity gradients. For example, the positive correlation coefficient between TOC and beta diversity of abundant taxa was greater than that of other environmental factors in LS samples. In the MS and HS groups, temperature and DIP were identified as the strongest factors influencing the beta diversity of abundant species (Supplementary Table S5). Additionally, the βNTI of the abundant subcommunity showed a significant association with DIP, DO and TOC. The abundant subcommunity assembly was primarily influenced by pH and TOC in the LS samples, while in the MS and HS groups, it was affected by TP and DIP, respectively (Supplementary Table S6). In the rare subcommunity, beta diversity in the LS group was mainly influenced by DO (Supplementary Table S5). Furthermore, similar to the abundant taxa, βNTI in rare taxa were primarily influenced by DIP, and the affect of DIP on βNTI was greater in rare biosphere than in the abundant biosphere. This study also found that βNTI was more strongly influenced by TP only in the MS group than in the abundant taxa, whereas in the LS and HS samples, the influence of pH and DIP on βNTI was weaker in the rare taxa than in the abundant taxa (Supplementary Table S6).

**Figure 6:**
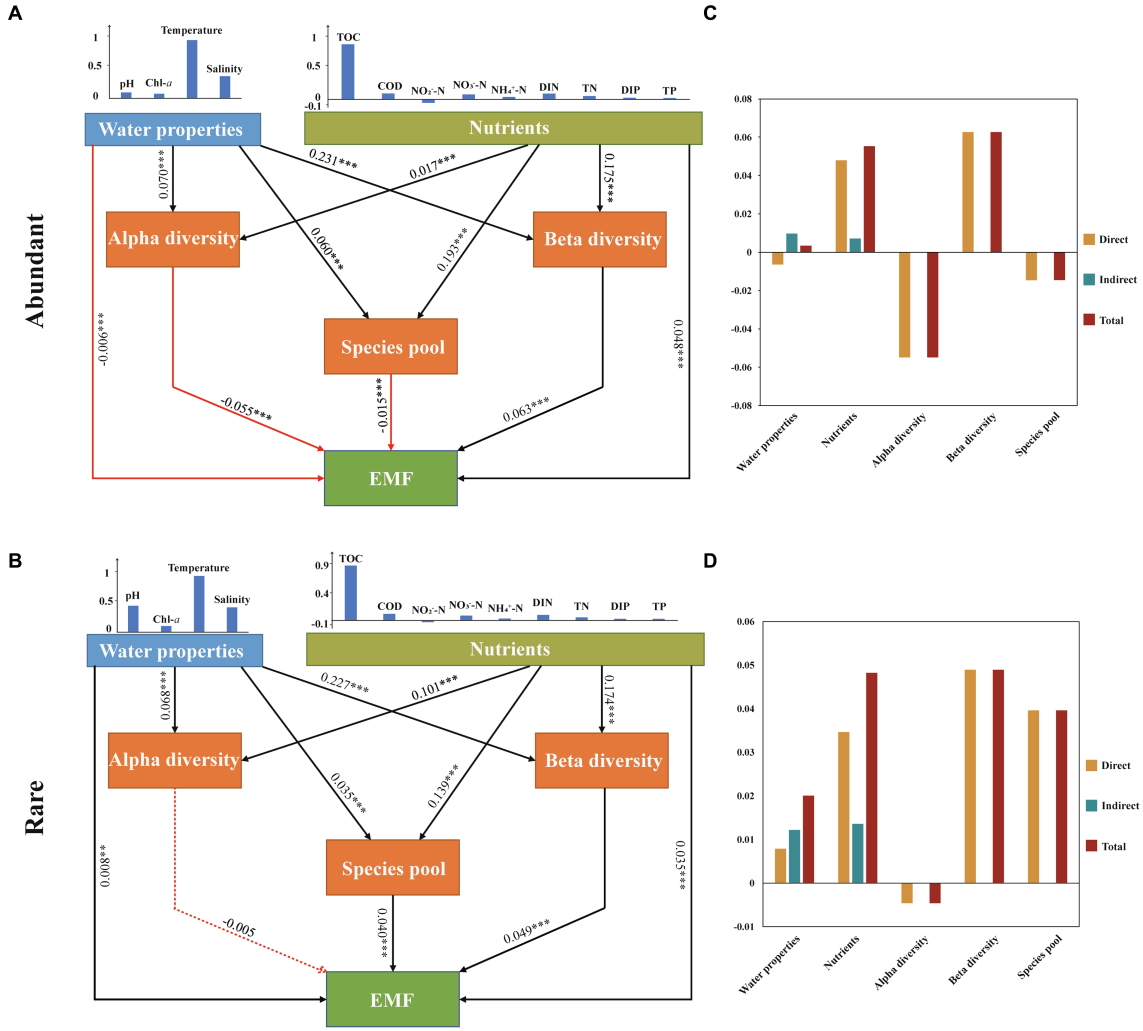
Partial least-squares path modelling (PLS-PM) analysis showing the multiple effects of environmental (water properties and nutrient parameters) factors and biotic (bacterioplankton community diversity and species pool) factors on EMF in marine ecosystems and the path coefficients between different factors and their corresponding latent variables of abundant **(A)** and rare **(C)** and their standardized direct, indirect and total effects of abundant **(B)** and rare **(D)**. Water properties include temperature, salinity, pH, and Chl-*a*. Nutrients include C (TOC, COD), N (NO_3_^−^-N, NO_2_^−^-N, NH_4_^+^-N, DIN, TN), and P (DIP, TP). Red arrows represent negative correlations, and blue arrows represent positive correlations. The solid arrow represents significant correlations (the number is the *R* value; the asterisk indicates the *p* value: *p < 0.05, **p < 0.01, and ***p < 0.001), and the dotted arrow represents nonsignificant correlations (*p* > 0.05).

In this study, we selected PLS-PM to investigate the complex relationships among water properties (WP), nutrients, microbial diversity (alpha and beta diversity), species pool, and EMF (Figure 6). In the abundant subcommunity, PLS-PM results revealed that WP had the strongest impact on alpha and beta diversity, exhibiting a significant positive correlation (*p* < 0.001). Among these factors, temperature emerged as the most critical variable influencing WP, whereas nutrients primarily exerted a significant influence on variations in the species pool through TOC (*p* < 0.001). Moreover, EMF were found to be primarily significantly positively regulated by beta diversity and nutrients (*p* < 0.001). In the rare subcommunity, PLS-PM results revealed that nutrients had the greatest significant impact on the alpha diversity and species pool of rare taxa (*p* < 0.001), with TOC being the most important variable influencing nutrients. WP was found to be the most significant variable for the beta diversity of rare taxa (*p* < 0.001), while the alpha diversity of taxa showed a nonsignificant correlation with EMF (*p* > 0.05). Additionally, temperature was an important parameter influencing EMF directly through beta diversity in both abundant and rare subcommunities compared to other factors (Figure 6).

## 4 Discussion

Studies suggested that under long-term environmental disturbance in estuaries, the nutrient cycle process is complex, resulting in a significant linkage between the structure and distribution of the microbial community and the changes in the surrounding environment (Wankel et al., 2011; Liu et al., 2014; Jeffries et al., 2016; Kieft et al., 2018). In this study, the main bacterial phylum in the abundant and rare taxa was Proteobacteria in the sampling region (Figure 1). The distribution pattern of Proteobacteria described here is consistent with previous studies in the Amazon River Estuary (Santos-Júnior et al., 2020), the Pearl River Estuary (Mai et al., 2020) and the Yellow River estuary (Wang et al., 2021). Proteobacteria not only exhibit a broad spectrum of metabolic diversity (e.g., photosynthetic metabolism, sulfate reduction, nitrogen metabolism) but also fulfil critical ecological roles, such as the decomposition of organic matter within the carbon cycle and the biodegradation of contaminants. These roles make the Proteobacteria essential in nutrient cycling processes, especially in estuarine ecosystems (Campbell et al., 2006; Hügler and Sievert, 2011; Kern et al., 2011; Chen et al., 2022). Similar to previous studies, we also found that the relative abundance of Proteobacteria increases with the salinity gradient in the surface water of estuarine environments (Tang et al., 2017; Mai et al., 2020; Santos-Júnior et al., 2020). In contrast, we also noted that the relative abundance of Bacteroidetes decreased from the river to the ocean. A previous study in Mediterranean Sea highlights that Bacteroidetes were more likely to survive in eutrophic environments (Pinhassi et al., 2004). Betaproteobacteria have been considered dominant in freshwaters and are an ecological indicator with high nitrogen content (Pinhassi et al., 2004; Yang et al., 2019). Overall, these results suggest that bacterial community is potential indicator of ecological resilience and water quality in estuarine systems.

The diversity characteristics of abundant and rare microorganisms play dominant roles in nutrient cycling, which is essential for assessing ecosystem functions (Xu et al., 2021; Du et al., 2024). The rare taxa showed higher alpha diversity than the abundant taxa, which is consistent with the finding of another study that found that rare species in the estuarine waters of the Perl River exhibit greater diversity than abundant species (Zhang et al., 2021). In this study, we also discovered that the alpha diversity of rare taxa exhibited larger interannual variation in HS samples than in the other samples. In addition, we noted that the species pool of the rare biosphere exhibited less variation than that of the abundant biosphere. This might be attributed to the result of the rare taxa fitness trade-offs, and the rare biosphere improves stress resistance at a lower growth rate under environmental disturbance (Jousset et al., 2017). Recent investigations have also indicated that owing to the immigration and emigration of rare species and the gradual recovery of their inhabitants densities (Jia et al., 2018; Xue et al., 2018), rare taxa may provide resilience or resistance abilities to the ecosystem (Gomez-Alvarez et al., 2016). In addition, several environmental factors were found to strongly affect the alpha diversity of different subcommunities (Supplementary Table S4). In agreement with previous researches (Campbell et al., 2011; Liu et al., 2015; Wan et al., 2021; Ren et al., 2022), both abundant and rare bacterial taxa exhibited sensitivity to distinct environmental factors. Our study reveal that the alpha diversity of both abundant and rare taxa was significantly influenced by multiple factors (Figure 6). These results highlight the different ecological adaptations of abundant and rare bacterial subcommunities to environmental disturbance and suggested that carbon compounds primarily affected abundant bacteria in Sanniang Bay. Accumulating evidence shows that the bacterial community can regulate nutrient cycling processes in aquatic ecosystems (Arrigo, 2005; Hutchins et al., 2009; Damashek and Francis, 2018; Cui et al., 2019; Baker et al., 2021), and enhance EMF (Winfree et al., 2015; Banerjee et al., 2018; Wan et al., 2021; Zhang et al., 2022). Studies exhibit that abundant and rare bacterial alpha-diversity have different significant contributions to EMF in various soil ecosystems (Winfree et al., 2015; Banerjee et al., 2018; Wan et al., 2021; Zhang et al., 2022). Our study found that there was no significant relationship between the EMF and the alpha diversity of abundant and rare taxa (Figure 3). These results suggest that due to estuaries being subject to unpredictable variations in salinity and water movement (Wang et al., 2017), abundant and rare bacteria have unique EMF in estuarine ecosystem.

Interestingly, our findings suggested that the beta diversity of rare taxa has a more intense promotional effect on EMF compared to abundant taxa (Figure 4). This result indicating that enhancement of beta diversity in rare biosphere may lead to functional redundancy (Grman et al., 2018). Given that the connection between EMF and its stability (Soliveres et al., 2016), rare species possess analogous functional capabilities to ensure the continuity of ecosystem functionality. The contributions of species pools containing both abundant and rare taxa to EMF, particularly estuary ecosystems, have not been the subject of many studies.

Our findings revealed that the species pool of abundant taxa exhibits a significant decreasing trend with the increase of EMF (Figure 5). Rare species may be more susceptible to environmental perturbation, as they have more pronounced ecological niche specificity (Zhang et al., 2022), while typically abundant taxa exhibit significant stable contributions in nutrient cycling within the ecosystem (Liu et al., 2015) and always higher competition potential and growth rate than rare taxa (Campbell et al., 2011). The size of abundant taxon species pools is more important to EMF. Previous study discovered that the richness of bacterial communities in the soil drives the diversity of soil ecological functions (Delgado-Baquerizo et al., 2017). But, in this study, the MNI tends to decrease as the size of the rare taxa species pool increases (*p* > 0.05). This result provides new insights into the association between microbial diversity and EMF in estuarine ecosystem.

A crucial aspect of revealing ecosystem function is to assess the relative importance of deterministic and stochastic processes to community assembly (Mokany et al., 2013; Mori et al., 2018). Similar to a previous study (Wang et al., 2021), we also confirmed that deterministic processes govern community assembly in coastal zone. In the present study, we discovered that in the studied region, deterministic assembly dominated both the rare and abundant bacterial subcommunities (Figure 2). As in previous studies (Liu et al., 2015; Li et al., 2021; Zhu et al., 2023), we noticed that the rare taxa were more affected by determinism than the abundant taxa. Rare taxa often exhibit narrower niche breadths, lower competitive abilities, and slower growth rates (Reveillaud et al., 2014; Ren et al., 2022). Furthermore, the enhancement of deterministic processes indicates that the shaping effect of environmental stress on microbial communities increases with the level of stress (Wang et al., 2023). Based on Mantel test, we found that the rare subcommunity βNTI in estuary-ocean ecosystems showed a stronger correlation with dissolved inorganic phosphorus (DIP) and total phosphorus (TP) than other variables (Supplementary Table S6). Phosphorus is identified as the primary limiting nutrient for ecosystem primary productivity (Peng et al., 2021) and its limitation can be regulated by bacteria to influence oceanic nutrient flux (Kritzberg et al., 2010). Therefore, it is an important factor influencing rare subcommunity structure and functional differentiation (Qin et al., 2020). DIP, DO, and TOC had significant effects on the succession of the abundant subcommunity. Different environmental factors have been found to determine the assembly of abundant and rare subcommunities in distinct habitats, such as temperature and pH, which control how rare and abundant bacterial subcommunities assemble in agricultural ecosystems (Jiao and Lu, 2020). Salinity and spatial factors significantly effected community turnover of abundant and rare bacterioplankton in Shenhu Bay, Dongshan Bay and Beibu Gulf (Mo et al., 2018). These results suggested that environmental filtering was a major factor in selecting microbial species and driving the assembly process in aqueous ecosystem (Ren et al., 2022).

Given that the environment can influence the diversity of different microbial subcommunities and their influences on EMF, we constructed a PLS-PM model (PLS-PM) to explore the interrelationships among WP, nutrients, bacterioplankton diversity (alpha and beta diversity), species pool, and EMF. In comparison to nutrients, WP (e.g., temperature) exerts a greater impact on the beta diversity of rare and abundant bacterioplankton taxa. The research conducted by Geng et al. (2022) indicates that environmental factors, including water temperature, significantly contribute to the beta diversity of bacterioplankton, consistent with the findings of this study. WP typically exhibits higher spatial and temporal variability compared to nutrients. This variability can lead to changes in community composition and beta diversity. Our results proved that the beta diversity of abundant and rare subcommunities was a better driver in EMF than alpha diversity. This finding is in line with the idea that microbial beta diversity is the strongest positive predictor of soil nutrient cycling processes (Xu et al., 2021). Differences in structure and metabolic functions among different species can give rise to their varying roles within the EMF (He et al., 2009). Due to the differential response capabilities of different species towards various environmental factors, beta diversity-rich communities can contribute to a greater range of EMF, particularly those associated with nutrient cycling. We further observed that, compared to abundant taxa, the beta diversity of rare taxa had a more significant impact on EMF. Due to the lower abundance of rare taxa, the variation in their beta diversity represents the presence or absence of unique species that may provide specific ecosystem services or participate in complex ecological processes, thus filling functional gaps and enhancing EMF (Jiao et al., 2017; Mori et al., 2018; Zhang et al., 2020; Xiong et al., 2021). Furthermore, we found that the relationship between beta diversity and EMF was mediated by temperature and TOC. Adhikari et al. (2019) posits that temperature is often linked to bacterial biodiversity, as it is directly related to metabolic rates and the affinity of bacteria for available substrates. Additionally, TOC is generally recognized as a crucial factor influencing microbial diversity variation and phylogenetic relationships within ecosystems, thereby being extensively reported as a primary determinant of microbial communities in global marine ecosystems (Li et al., 2020).

## 5 Conclusion

Our study identifies both abundant and rare biospheres in a subtropical estuary. Rare biospheres exhibit higher diversity and are more important for ecosystem functions than abundant biospheres. Deterministic processes dominate in the community assembly of abundant and rare taxa. The bacterial beta of rare taxa has a significant impact on EMF in the subtropical estuary, and different abiotic factors were established to affect EMF of abundant and rare taxa. Unexpectedly, EMF were strongly linked to the species pool of the abundant subcommunity. Overall, the results obtained in this study improve our understanding of how abundant and rare taxa impact EMF in estuary ecosystems and advance our comprehension of ecological diversity and function.

## Data availability statement

The datasets presented in this study can be found in online repositories. The names of the repository/repositories and accession number(s) can be found at: https://www.ncbi.nlm.nih.gov/genbank/, PRJNA971362.

## Author contributions

SY: Supervision, Writing – review & editing, Conceptualization, Methodology, Validation. QH: Writing – review & editing. NL: Supervision, Writing – review & editing, Investigation, Visualization. PW: Conceptualization, Methodology, Writing – review & editing. HZ: Conceptualization, Methodology, Writing – review & editing. QC: Supervision, Writing – review & editing. XQ: Formal analysis, Investigation, Supervision, Writing – review & editing. JH: Investigation, Supervision, Writing – review & editing. XL: Writing – review & editing. NL: Writing – review & editing. GJ: Formal analysis, Visualization, Writing – review & editing. KD: Writing – review & editing. TZ: Conceptualization, Data curation, Formal analysis, Investigation, Methodology, Visualization, Writing – original draft, Writing – review & editing.
