# Supplementary material for: Rare subcommunity maintains the stability of ecosystem multifunctionality by deterministic assembly processes in subtropical estuaries
Source: Front Microbiol. 2024 Apr 19;15:1365546. doi: 10.3389/fmicb.2024.1365546 (PMC11066265; doi:10.3389/fmicb.2024.1365546)
Supplement: Supplementary file 1 [file Table_1.DOCX]

**Supporting Information-1 (Figure S1-S2)**

**Rare subcommunity maintains the stability of ecosystem multifunctionality by deterministic assembly processes in subtropical estuaries**

**Shu Yang^1,2†^, Qinghua Hou^1†^, Nan Li^1^, Pengbin Wang^3^, Huaxian Zhao^2^, Qingxiang Chen^1^, Xinyi Qin^2^, Jiongqing Huang^4^, Xiaoli Li^4^, Nengjian Liao^5^, Gonglingxia Jiang^1^, Ke Dong^6^, Tianyu Zhang^1^***

^1^ Key Laboratory of Climate, Resources and Environment in Continental Shelf Sea and Deep Sea of Department of Education of Guangdong Province, Department of Oceanography, Key Laboratory for Coastal Ocean Variation and Disaster Prediction, College of Ocean and Meteorology, Guangdong Ocean University, Zhanjiang 524088, China

^2^ Key Laboratory of Environment Change and Resources Use in Beibu Gulf, Ministry of Education (Nanning Normal University), Nanning, Guangxi, 530001, China

^3^ Key Laboratory of Marine Ecosystem Dynamics, Second Institute of Oceanography, Ministry of Natural Re-sources, Hangzhou 310012, China

^4^ School of Agriculture, Ludong University, Yantai 264025, China

^5^ College of Environmental Science and Engineering, Guilin University of Technology, Guilin 541004, China

^6^ Department of Biological Sciences, Kyonggi University, 154-42, Gwanggyosan-ro, Yeongtong-gu, Suwon-si, Gyeonggi-do 16227, South Korea; Republic of Korea

*** Correspondence:**Corresponding Author: Tianyu Zhang

zhangty@gdou.edu.cn

†Shu Yang and Qinghua Hou contributed equally to this work and share first authorship


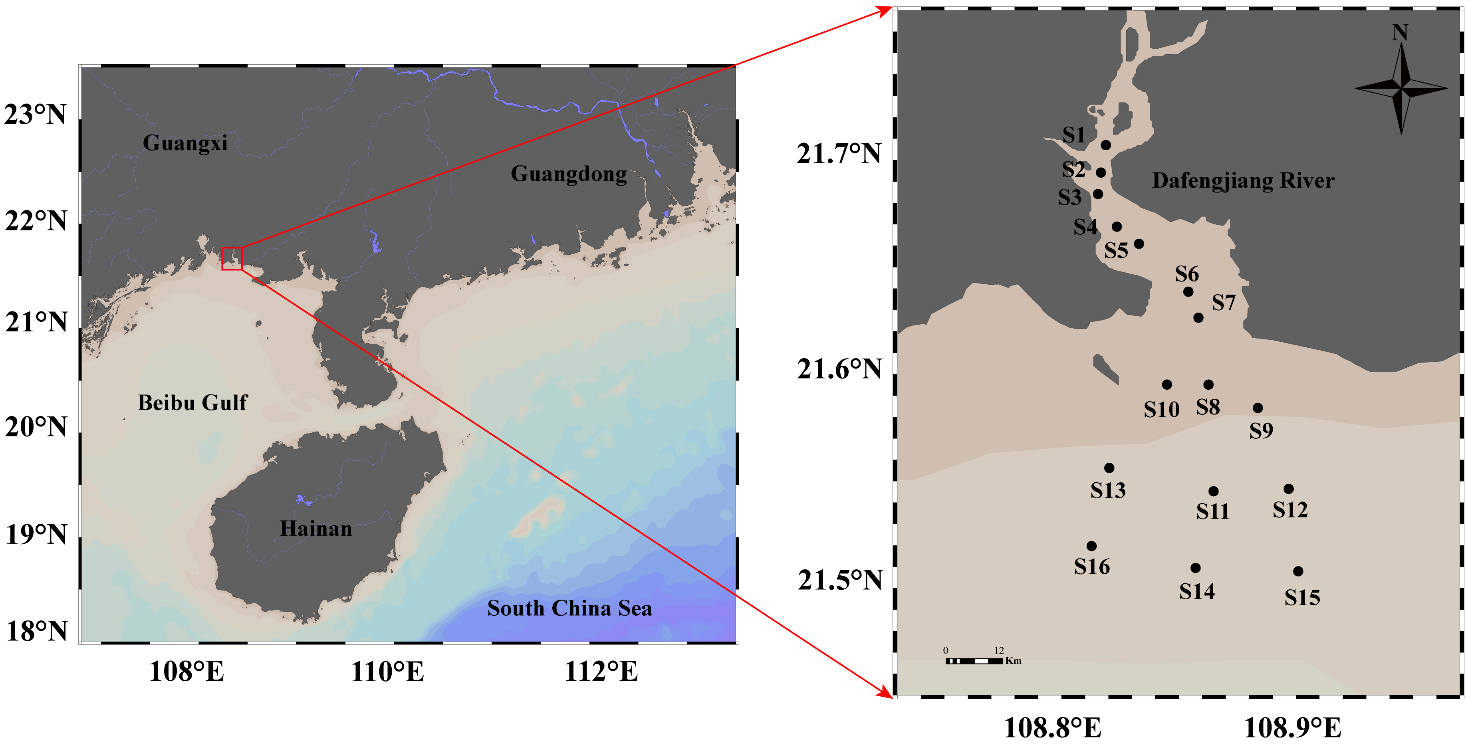


**Figure S1:** Geographic map of the fifteen sampling sites for marine bacterioplankton in the estuary area of Sanniang Bay, Guangxi Zhuang Autonomous Region, China.


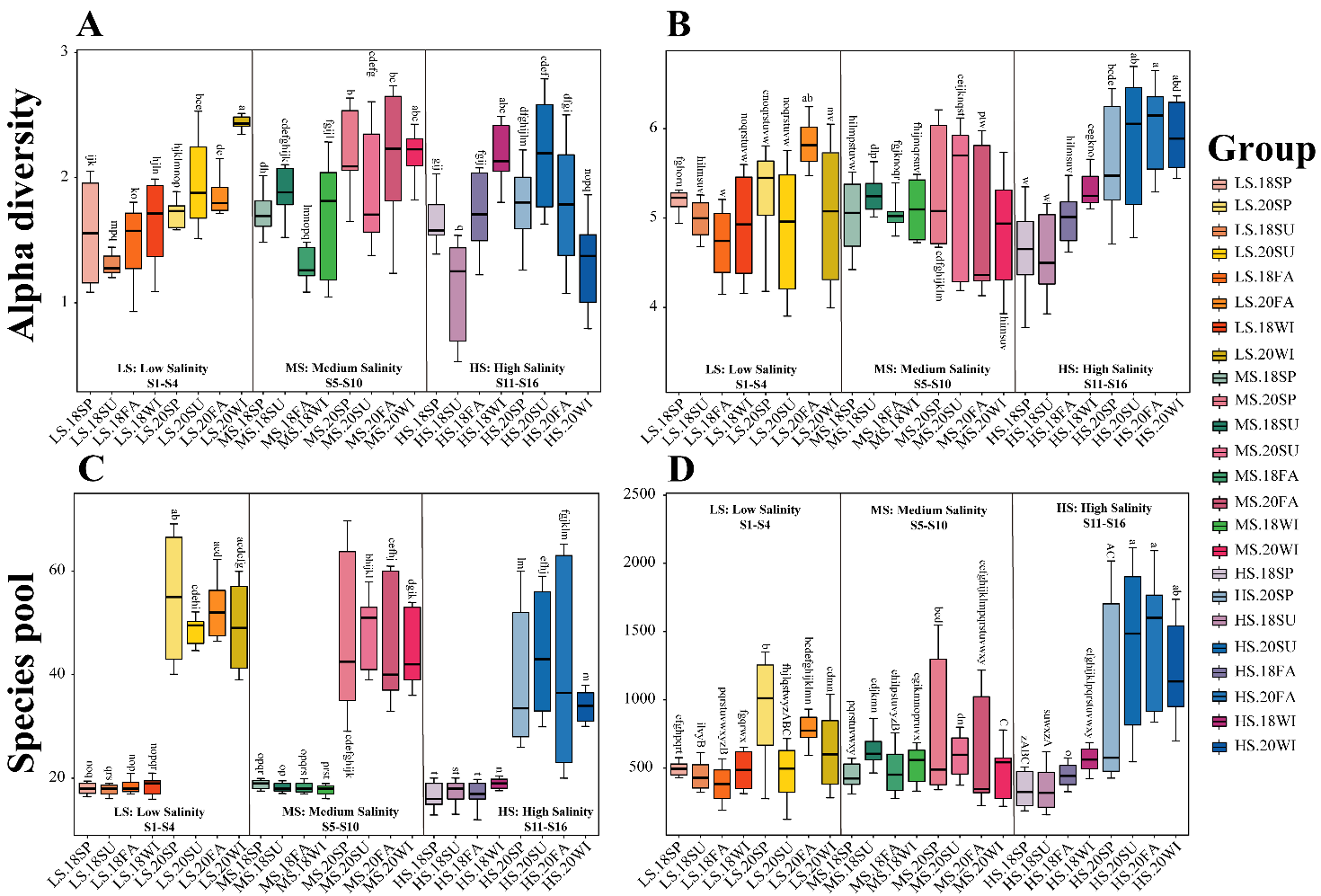


**Figure S2:** The alpha diversity and species pool indices of abundant (A, C) and rare (B, D) marine bacterioplankton taxa from different seasons and salinity gradients (LS: low salinity; MS: medium salinity; HS: high salinity. SP: Spring; SU: Summer; FA: Fall; WI: Winter).
