# Supplementary figures and images for: Rare subcommunity maintains the stability of ecosystem multifunctionality by deterministic assembly processes in subtropical estuaries
Source: Front Microbiol. 2024 Apr 19;15:1365546. doi: 10.3389/fmicb.2024.1365546 (PMC11066265; doi:10.3389/fmicb.2024.1365546)

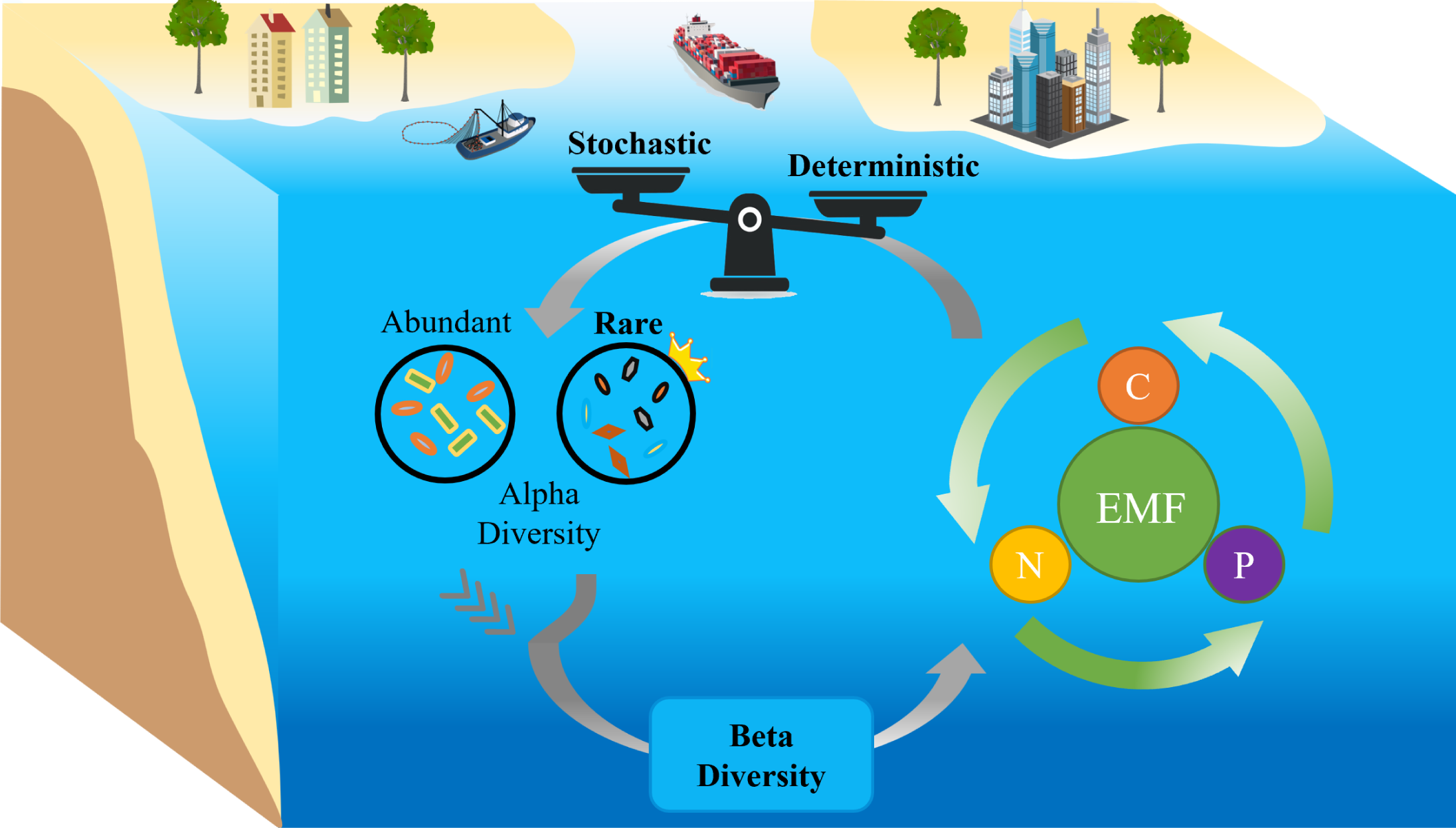

Supplement: Supplementary file 3 [file Image_1.TIF]
